# Precomputed low-frequency lighting in cinematic volume rendering

**DOI:** 10.1371/journal.pone.0312339

**Published:** 2024-10-21

**Authors:** Yuliang Yuan, Jinzhu Yang, Qi Sun, Yan Huang

**Affiliations:** School of Computer Science and Engineeing, Northeastern University, Shenyang, Liaoning, P. R. China; Jinka University, ETHIOPIA

## Abstract

Cinematic Rendering (CR) employs physical models such as ray tracing and global illumination to simulate real-world light phenomena, producing high-quality images with rich details. In the medical field, CR can significantly aid doctors in accurate diagnosis and preoperative planning. However, doctors require efficient real-time rendering when using CR, which presents a challenge due to the substantial computing resources demanded by CR’s ray tracing and global illumination models. Precomputed lighting can enhance the efficiency of real-time rendering by freezing certain scene variables. Typically, precomputed methods freeze geometry and materials. However, since the physical rendering of medical images relies on volume data rendering of transfer functions, the CR algorithm cannot utilize precomputed methods directly. To improve the rendering efficiency of the CR algorithm, we propose a precomputed low-frequency lighting method. By simulating the lighting pattern of shadowless surgical lamps, we adopt a spherical distribution of multiple light sources, with each source capable of illuminating the entire volume of data. Under the influence of these large-area multi-light sources, the precomputed lighting adheres to physical principles, resulting in shadow-free and uniformly distributed illumination. We integrated this precomputed method into the ray-casting algorithm, creating an accelerated CR algorithm that achieves more than twice the rendering efficiency of traditional CR rendering.

## 1 Instructions

In 2016, Drebin et al. [1] introduced cinematic rendering (CR) to visualize medical images and assist doctors in disease diagnosis and treatment planning. CR is generally utilized for offline rendering, where high-quality output is prioritized, and rendering time is less critical. However, in the medical field, doctors need both high-quality and real-time performance, which imposes stringent demands on CR algorithms.

The primary approach for CR involves using path tracing algorithms to simulate light propagation within the data and a physics-based global illumination model to replicate the physical interactions of light with objects. Path tracing algorithms often employ Russian roulette to calculate the state of light propagation randomly. This technique can introduce noise and waste computing resources, reducing rendering efficiency. Although the physics-based global illumination model accurately simulates shadows and multiple scattering to achieve natural and precise rendering effects, its inefficiency makes it unsuitable for real-time requirements.

To address these issues, we have designed a real-time CR algorithm based on photon mapping and the ray-casting algorithm. Our rendering algorithm divides light into low-frequency ambient light and direct light. For ambient light, we build a physics-based precomputed global ambient illumination field. During the rendering process, this global ambient illumination field is combined with direct light to simulate the physical effects of environmental light and light sources, achieving realistic rendering effects. Since the global ambient illumination field is precomputed, our algorithm can achieve extremely high efficiency during the rendering process without reducing the resolution during camera transformations.

Our main contributions to this work are:

1. We propose a precomputed low-frequency lighting method based on photon mapping, which achieves sufficient ambient light rendering for semi-transparent volume data. This precomputed method is applied in the physical rendering algorithm, significantly reducing rendering time while maintaining a comparable level of rendering quality.
2. We implement physical rendering in the ray-casting algorithm, achieving smooth and realistic rendering outcomes(CR).

## 2 Background

Medical images usually come from large medical devices such as PET (Positron Emission Tomography), CT (Computed Tomography), and MR (Magnetic Resonance Imaging). Three-dimensional visualization of the image data collected by these devices can help doctors achieve enhanced diagnosis. Early on, due to hardware limitations, three-dimensional visualization typically relied on empirically-based models to calculate object lighting phenomena. Commonly used lighting models included the Lambert model, the Phong lighting model (Mukunoki and Takahashi [2]), and the Blinn-Phong improved lighting model. These models primarily used local data features, such as gradients, to simulate the details and occlusion relationships of human tissue. However, because these features are local, they could not achieve physically-based natural and realistic rendering. The rendering algorithms using these lighting models are known as volume rendering algorithms, first proposed by Drebin et al. [3] in the late 1980s. Common algorithms for volume rendering include ray casting, splatting, and shear-warp. While these algorithms offer high rendering efficiency, they cannot achieve realistic rendering due to the limitations of the lighting models.

With the advancement of hardware technology, physically-based global lighting model rendering has become possible. Following the introduction of physically-based cinematic rendering, Marwen Eid [4] illustrated its potential advantages and applications in CT in 2017. Since then, cinematic rendering technology has been extensively researched and primarily used for disease diagnosis. For example, Chul et al. [5] used cinematic rendering to diagnose and detect pancreatic cancer, and Rowe et al. [6] applied it for CT evaluation of musculoskeletal trauma. Since the outbreak of COVID-19, Necker et al. [7] have performed chest CT image reconstruction of SARS-CoV-2 pneumonia using cinematic rendering.

The CR algorithm originates from offline physical rendering used in movie scenes, characterized by long rendering times and high realism. The CR algorithm adapts this physical rendering approach to the field of medical imaging, where it employs transfer functions to perform physically-based volume rendering of medical data. The rendering results must meet the real-time diagnostic needs of physicians. CR algorithms generally employ path tracing methods to construct physically-based global illumination models, simulating how light passes through objects in a physically accurate manner. However, the efficiency of path-tracingpath tracing is inherently low due to the need for full-path calculations. Additionally, Monte Carlo integration, which is widely used in path tracing, introduces issues such as slow convergence and noisy output images. Thus, achieving real-time rendering often requires a balance between rendering quality and efficiency.

## 3 Relate work

Appel et al. [8] first proposed the ray tracing algorithm; however, it was both inefficient and limited. Later, Kajiya et al. [9] introduced the path tracing algorithm, which helped to improve rendering efficiency. Following that, Veach et al. [10] proposed the bidirectional path tracing algorithm, which was capable of handling global illumination, reflection, and refraction, thus producing high-quality images. Nevertheless, this algorithm still did not meet real-time rendering requirements.

In order to enhance rendering performance for real-time applications, Salama [11] proposed a Monte Carlo-based ray tracing method, which improved performance by limiting light scattering on object surfaces. Yet, this method was unsuitable for rendering translucent objects. Similarly, Kroes et al. [12] further improved Monte Carlo ray tracing by developing an interactive method that supported multiple light sources and complex materials, leading to higher-quality rendering. Even so, the computational time for this method remained quite long.

To address the ongoing need for real-time rendering, Jensen et al. [13] introduced the photon mapping algorithm, which handled global illumination and indirect lighting with higher computational efficiency, although it required significant memory resources. While Progressive Photon Mapping [14] alleviated the memory issue, it still demanded considerable computational time due to the need for extensive photon statistics. Later, Kwon et al. [15] introduced a method to reduce the computation needed for photon mapping by using a light distribution template; however, the fixed template posed challenges for the flexible adjustment of transfer functions in volume rendering. More recently, Iglesias-Guitian et al. [16] proposed a real-time path tracing algorithm that achieved better rendering results with fewer samples per pixel (SPP). However, this algorithm suffered from noise and resource inefficiencies due to uniform random sampling.

Zhang et al. [17] implemented Precomputed Photon Mapping in Real-Time Volume Rendering, where photons were generated at the volume boundary. Yet, because the emission direction and the physical phenomena were random, the approach resulted in low efficiency and weak support for semi-transparent transfer functions.

Recently, deep learning techniques have gained momentum, with algorithms such as [18, 19] improving both rendering efficiency and image quality. Additionally, methods such as [20, 21] for SR and DOF are increasingly being used in rendering. However, these approaches require large training datasets that cannot accommodate all real-time rendering needs for multiple transfer functions. Moreover, the significant computational demands of deep learning algorithms make them difficult to apply in real-time rendering.

Due to frequent camera changes and the influence of translucent transfer functions, implementing a precomputed method that complies with physical laws in CR is complex, and there is little related research. The method proposed by Freude et al. [22] requires the extraction of isosurfaces, which remains similar to traditional polygon-based rendering.

## 4 Our algorithm

According to the rendering equation, we can divide the illumination into direct illumination *L*_0_, indirect illumination *L*_1_ after one reflection, and indirect illumination *L*_*n*_ after more reflections. The light can be written as Eq 1:

$$\begin{array}{c} L=L_{0}+\sum_{p=1}^{n}L_{p} \end{array}$$ (1)

$\sum_{p=1}^{n}L_{p}$ represents indirect illumination, which can be considered as ambient light. The purpose of our algorithm is to precompute the ambient light.

In volume rendering, the ambient light we precompute needs to be low-frequency lighting to contrast with the direct lighting *L*_0_ to form a direct shadow area. Since volume rendering involves frequent camera changes, obtaining the value of ambient light in real-time rendering independent of the current camera direction is necessary. However, as shown in Eqs 2 and 3:

$$\begin{array}{c} L_{1}=L_{c}*k_{d}*max\left(h\cdot v,0\right)\; \end{array}$$ (2)

$$\begin{array}{c} L_{all}\approx L_{c}*k_{d}\sum_{p=1}^{n}*max\left(h\cdot v,0\right) \end{array}$$ (3)

The conventional physical lighting algorithm needs to account for all the lighting in the hemisphere at the location, and it must be recalculated once the camera changes again.

We propose a solution to the above problem. Since the camera angle and illumination are strongly related, we abbreviate the illumination as Eq 4, where $\vec{D}$ is the parameter of the incident direction of the light. When precalculating the ambient light, the light source distribution is arranged according to spherical geometric symmetry, and the light source function *DF*(*φ*) is defined, where *φ* is the angle parameter. As shown in Eq 5, after the light source surrounds the volume data in a spherically symmetrical distribution, the influence of the angle is offset, and the illumination terms involved at any angle are roughly the same. When the light intensity of the light source is consistent, under the influence of multiple light sources, the result also tends to be a low-frequency distribution.

$$\begin{array}{c} L\left(x,\omega \right)=\sum_{p=1}^{n}f\left(x,\omega_{p},\omega \right)\vec{D}*\phi_{p}\left(x_{p},\omega_{p}\right) \end{array}$$ (4)

$$\begin{array}{c} L\approx \sum_{p=DF(0)}^{DF(4\pi r^{2})}f\left(x,\omega_{p},\omega \right)\phi_{p}\left(x_{p},\omega_{p}\right) \end{array}$$ (5)

**Fig 1:**
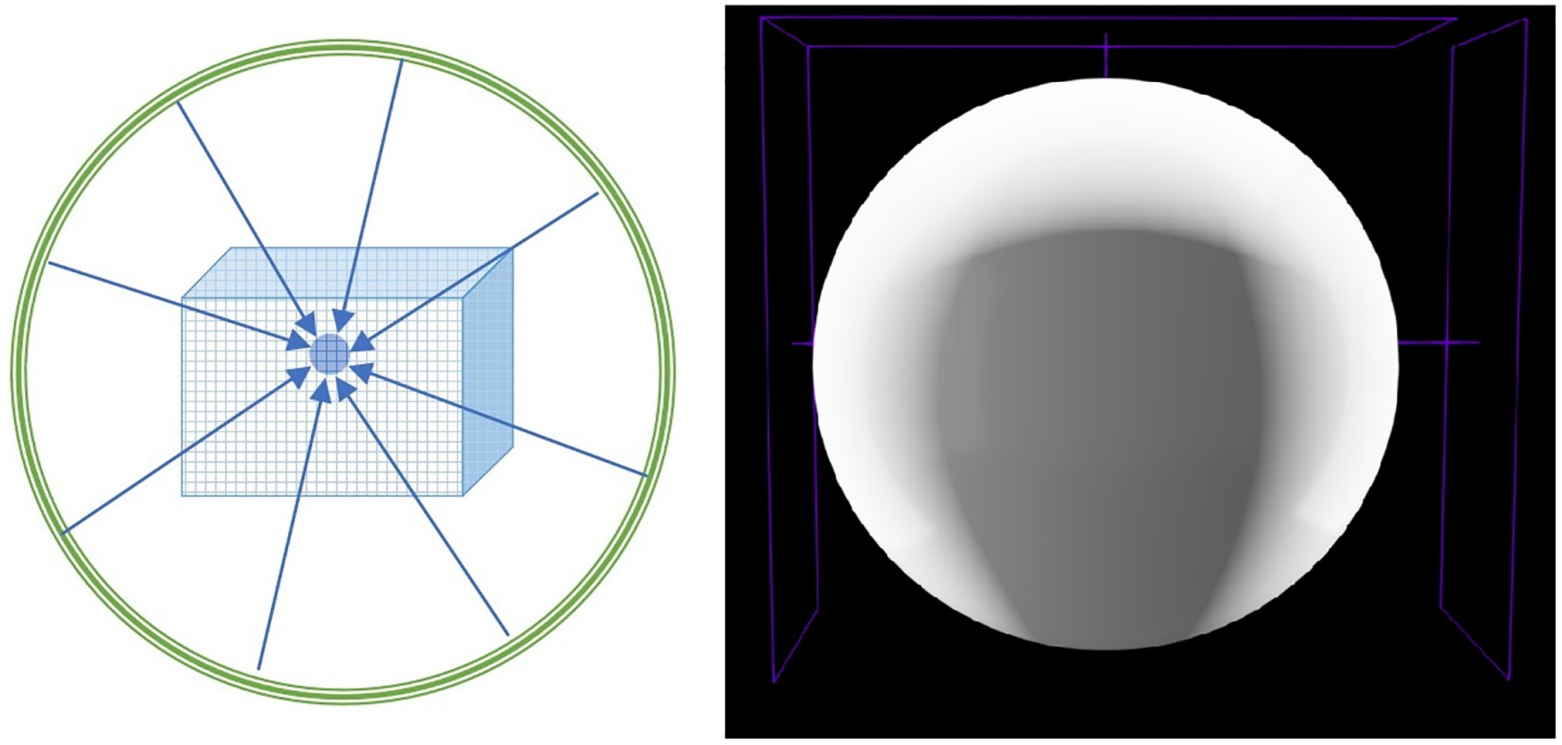
Illumination principle schematic diagram. On the right is a schematic of geometric rendering. To highlight the shadowed areas, three area light sources are used to achieve uniform illumination except for the shadow regions. If the fully covering multi-light source rendering from the left diagram is used, consistent lighting can be achieved regardless of the camera.

As shown in Fig 1, this diagram provides a visual explanation of our method. The rendering results for points under a spherical light source remain consistent, regardless of the camera angle.

According to the above content, Our algorithm is implemented in two parts, precompute the ambient light field and real-time fusion rendering.

In the precompute ambient light field, we construct a global ambient light field using the photon mapping method to simulate ambient light.

In the real-time fusion rendering phase, we use the ray-casting algorithm and a fusion equation that combines ambient light and transmitted light, which we defined, to perform fusion rendering.

### 4.1 Ambient light field

The ambient light must fully render all volume data voxels. If the lighting coverage is incomplete, there will be black non-rendering areas. It is crucial to ensure the light intensity is low-frequency; otherwise, there will be overexposure or underexposure. Additionally, lighting based on transfer functions will produce prominent shadow areas due to the occlusion of opaque voxels, causing insufficient rendering problems that must be avoided.

We use the following physical theorem to implement ambient light rendering: When light shines on an object, shadows are produced due to occlusion. When the distance between the light source and the occlusion object is constant, the size of the umbra is inversely proportional to the illumination angle and the area of the light source. The more light that comes from different directions, the smaller the umbra becomes. The umbra will disappear completely when the light source area is large enough. The shadowless lamp used in surgery utilizes this physical principle.

#### 4.1.1 Create multiple light sources

The light source function is defined as Eq 6 follows, where the calculated *P*_*ray*_ is the location of the light source, *R*_*center*_ is the center of the volume data, and *R*_*radius*_ is the maximum circumscribed sphere radius of the volume data:

$$\begin{array}{c} P_{ray}=R_{center}*+R_{radius}*\vec{D}\left(r,\theta ,\varphi \right) \end{array}$$ (6)

**Fig 2:**
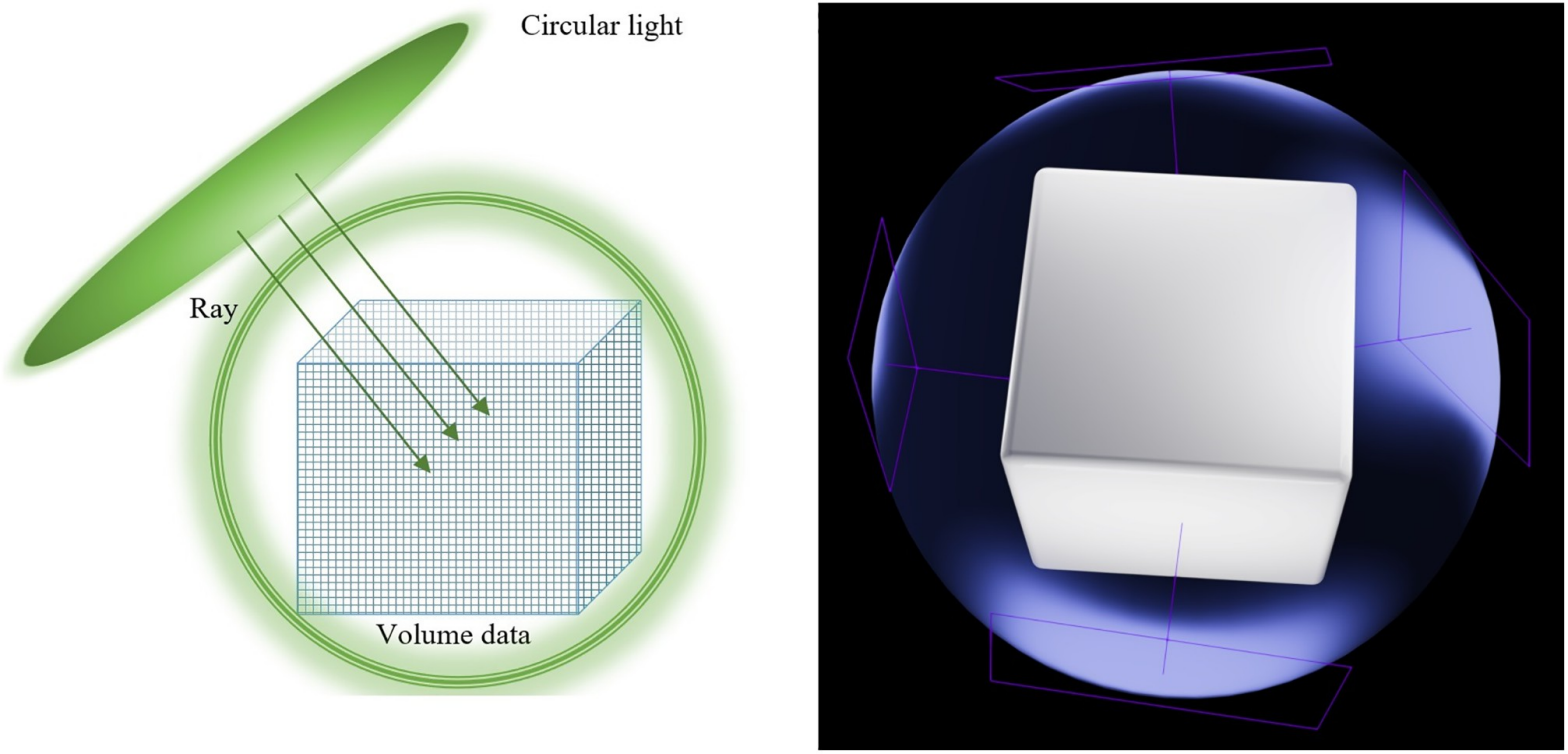
Circular light source diagram. The figure on the right is a schematic diagram of 3D rendering, showing that the four sets of lights can illuminate the corresponding areas. Ultimately, we used 72 sets of lights to render the data, achieving uniform illumination in the 3D space.

The direction of the light source is $\vec{L}=-\vec{D}$, The light source is circular with a radius of *R*_*radius*_, as shown in Fig 2. The light source must cover the entire volume data, so the radius of the light source must be larger than *R*_*radius*_. If the light source coverage is incomplete, there will be uneven lighting, which is unacceptable.

**Fig 3:**
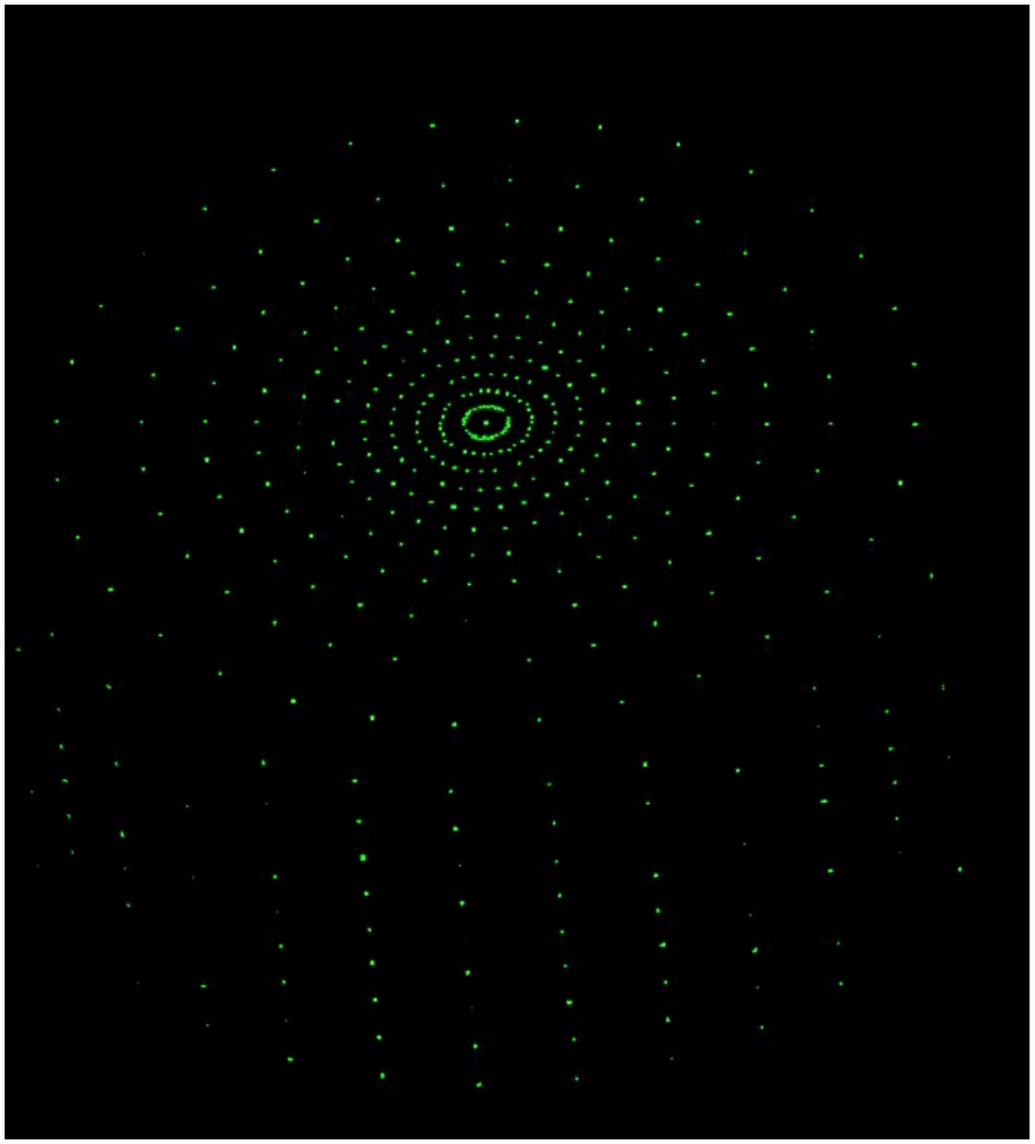
Ambient light diagram.

As shown in Fig 3, a spherical light source field surrounding the volume data is constructed on the periphery of the volume data according to Eq 6. Under this spherical light source distribution, based on the physical laws of shadowless lamps, the angle between the object and the light source loses its significance. As shown in Eq 5, the final ambient light rendering result is the integral of all light source renderings:

Define the global ambient light field *Vp* = {*Vpx*, *Vpy*, *Vpz*} to store ambient light. *Vpx*, *Vpy* are consistent with the pixel width and height of medical images, and *Vpz* is the height in pixels, wwhich can be calculated using the Eq 7

$$\begin{array}{c} Vpz=(Vz-1)*Ps \end{array}$$ (7)

where *Vpz* is the slice count of the volume data, and *Ps* is the pixel spacing of the volume data. The global ambient light field is consistent with the voxel size and resolution of the volume data.

Use the photon mapping algorithm to inject photons according to the method in Fig 3. This process mainly involves two parts: the photon energy equation and ambient light rendering.

#### 4.1.2 Photon energy equation

We define that light will reflect and transmit when passing through voxels. Light will only experience physical phenomena when passing through voxels whose transfer function transmittance *α* is not zero. We use Schlick’s approximation to calculate the reflectivity. For convenience in calculation, we define the light that is reflected and transmitted as the new light:

Define the input ligh *L*_*C*_, refract light *L*_*R*_, transmitt light *L*_*I*_ and current light *L*_*C*_ which writes into the Ambient Light Field:

$$\begin{array}{c} L_{T}=L*\left(1-\alpha \right) \end{array}$$ (8)

$$\begin{array}{c} L_{R}=L*F_{Schlick} \end{array}$$ (9)

$$\begin{array}{c} L_{C}=L-L_{R}-L_{I} \end{array}$$ (10)

$$\begin{array}{c} F_{Schlick}\left(h,v,F_{0}\right)=F_{0}+\left(1-F_{0}\right)*pow\left(\left(1-\left(h\cdot v\right)\right),5\right)) \end{array}$$ (11)

F0 is the Fresnel reflection factor material coefficient, which defines the ratio of refraction and reflection. The volume data’s current point gradient and camera direction can calculate (*h* ⋅ *v*).

#### 4.1.3 Ambient light rendering

**Fig 4:**
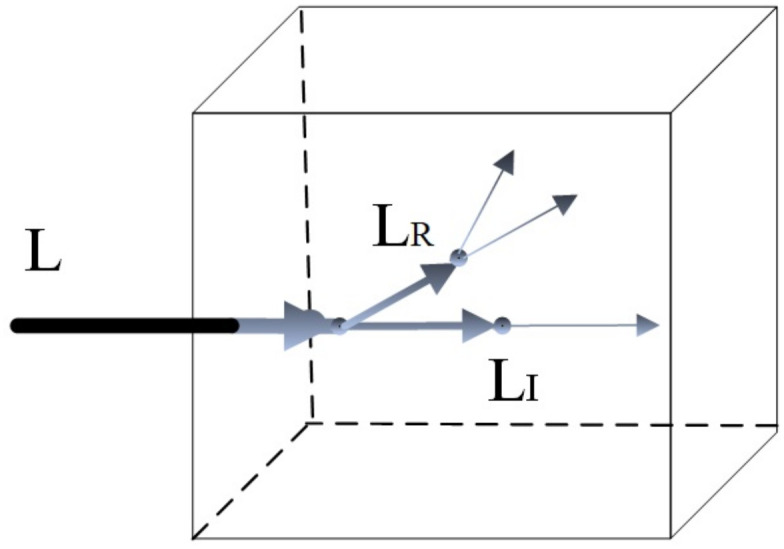
Light path diagram.

When the light passes through each point where *α* is not 0, the light is split into two rays of transmittion and refraction, as shown in Fig 4. The light is split step by step until the photon is less than *ϵ*, and *ϵ* is the minimum light intensity.

For each point in the ambient light field, the final value of the photon is the sum of all passing rays:

$$\begin{array}{c} L\left(x,y,z\right)=\sum_{p=1}^{n}L_{C}\left(x,y,z\right) \end{array}$$ (12)

After the precomputation, we get an ambient light energy field based on the transfer function, and the point value in the ambient light energy field represents the light intensity.

**Fig 5:**
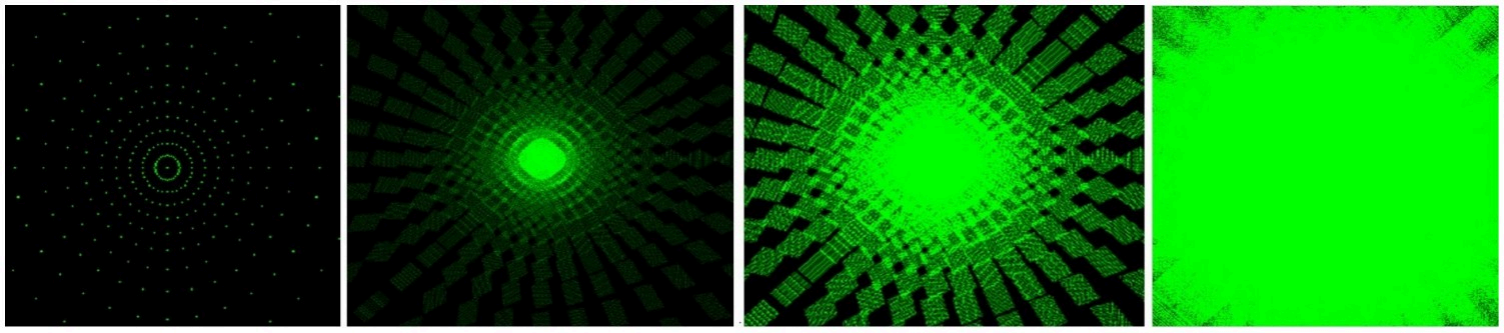
Light area change diagram.

**Fig 6:**
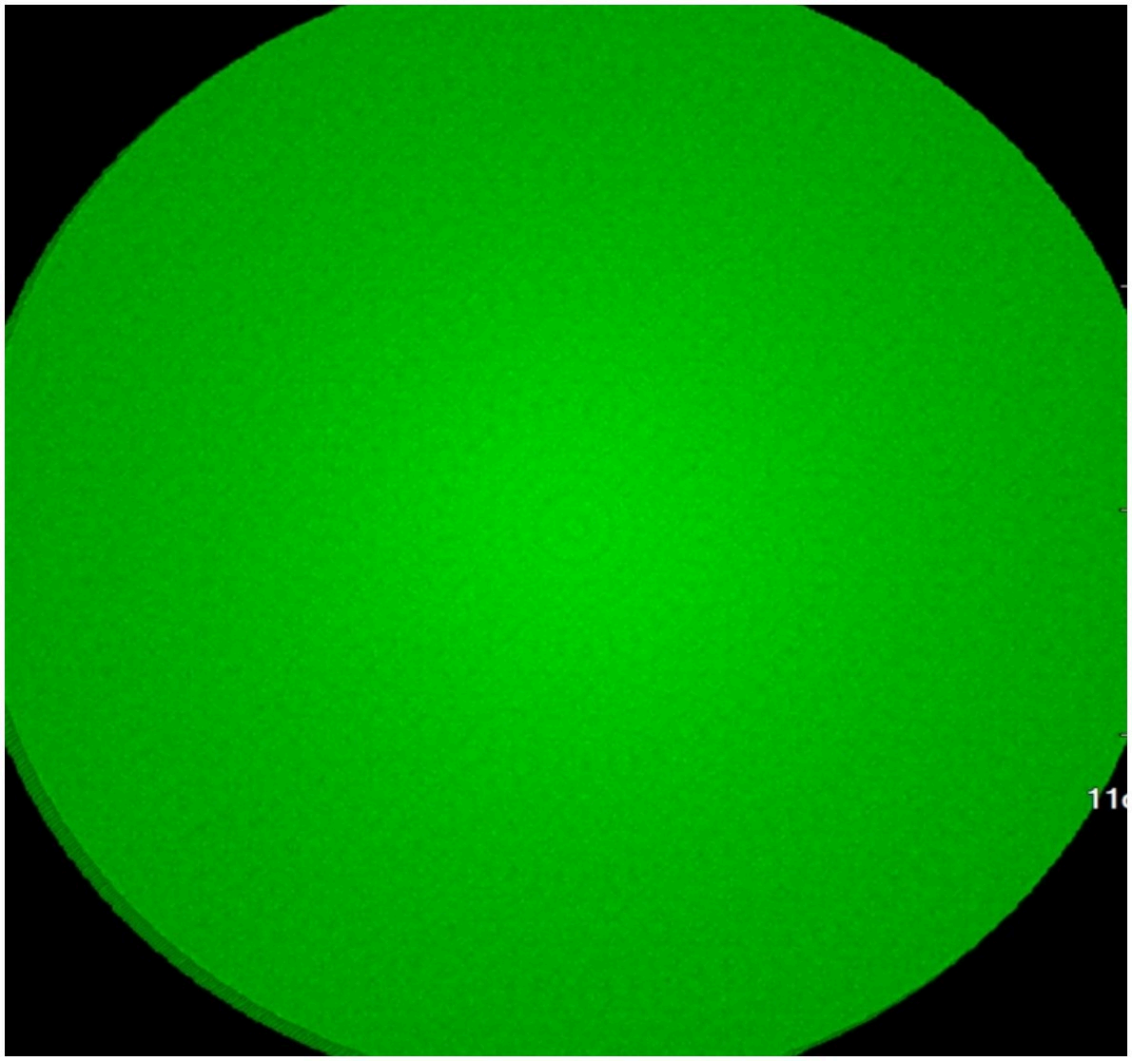
Ambient light rendering result diagram.

As shown in Fig 5, when all light sources have the same intensity, as the radius *R*_*radius*_ light sources continues to increase, the area of the light sources continues to increase, and the illumination gradually becomes uniform, and the black non-rendering area gradually decreases. The final result, shown in Fig 6, is the state of low-frequency full rendering.

### 4.2 Real-time rendering

We use the ray-casting algorithm to simulate transmitted light in real-time rendering. During the ray-casting process, the fusion rendering equation combines the transmitted light with the precomputed ambient light for each involved volume data point. The final rendering result is obtained by summing these contributions.

#### 4.2.1 Ambient light interpolation

Set the point *P*(*x*, *y*, *z*) in the volume data, and let the distance between point *P* and surrounding neighbor points be *D*_*i*_. The light intensity value of the neighboring point is *L*_*i*_. The ambient light interpolation equation is defined as Eq 13:

$$\begin{array}{c} L_{interp}\left(P\left(x,y,z\right)\right)=\frac{\sum_{p=1}^{n}\frac{1}{D_{i}}*L_{i}}{\sum_{p=1}^{n}\frac{1}{D_{i}}} \end{array}$$ (13)

**Fig 7:**
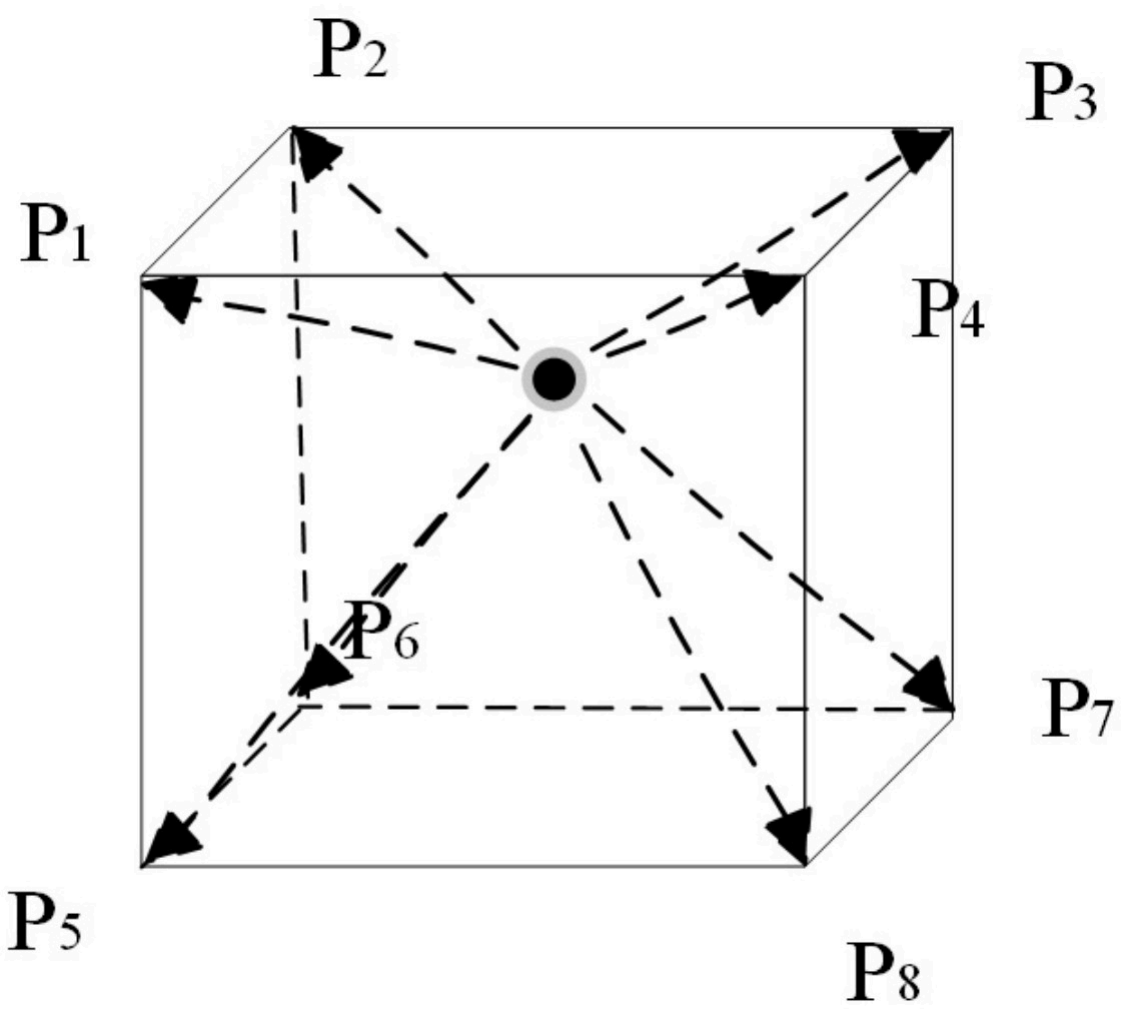
Ambient light interpolation diagram.

The neighboring points of point *P* are shown in the Ambient Light Interpolation diagram Fig 7.

**Fig 8:**
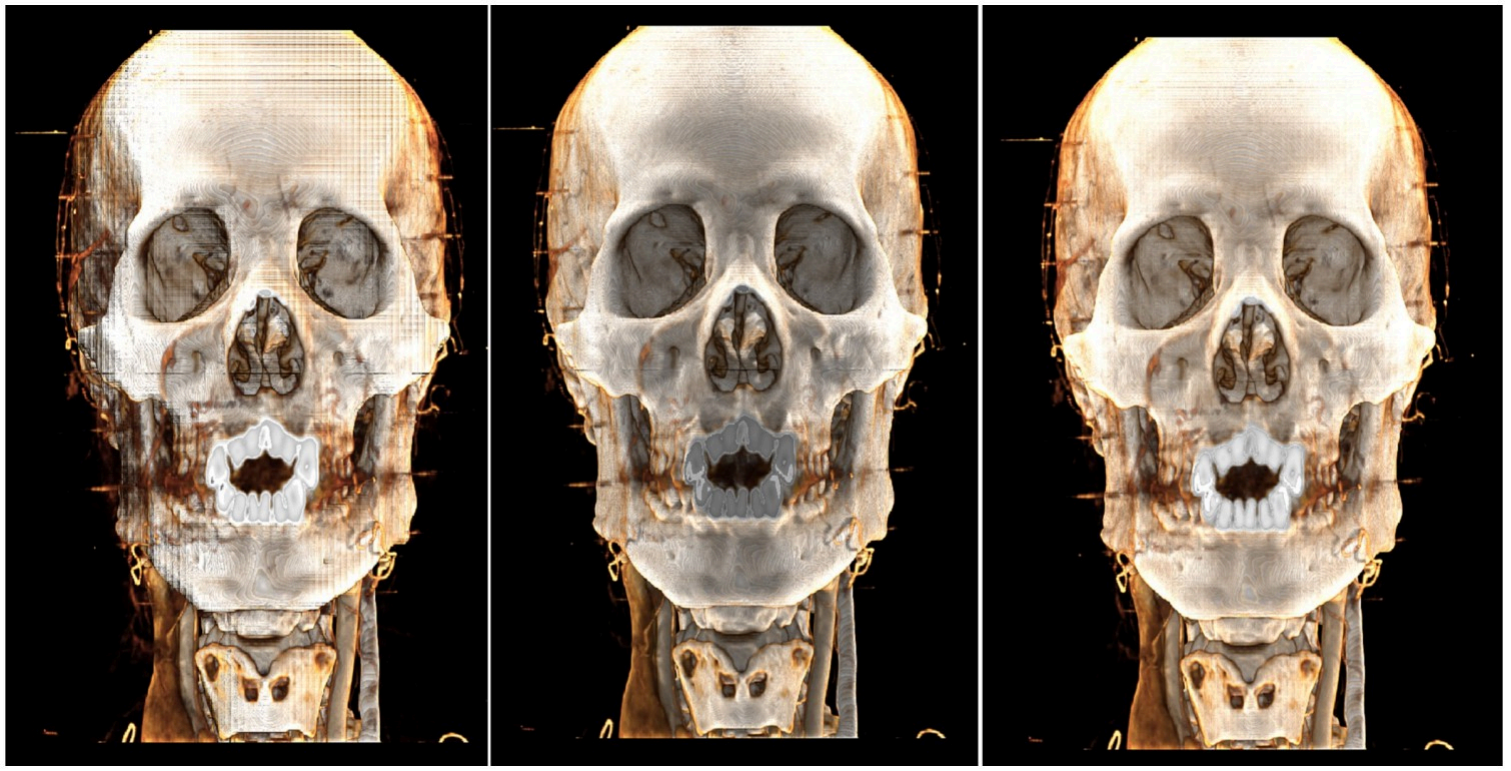
Ambient light comparison diagram.

The precomputed ambient light field is calculated with the transfer function as a parameter. As a result, the global light energy field is superficially distributed, with *L*_*i*_ = 0 points existing. As shown in Fig 8, the distance-based method can effectively avoid the situation where *L*_*i*_ = 0, ensuring accurate calculation results.

Distance interpolation based on inhomogeneous fields can produce artifacts on perfectly smooth planes. We can take Eq 13 as input and use beyond trilinear interpolation [23] to calculations or use the pre-integration [24] method in precomputation.

#### 4.2.2 Fusion rendering

Unlike the illumination model based on local features, since we have obtained the global ambient light field, there is no need to perform global feature calculations. We only fuse ambient and transmitted light using a linear model.

First, obtain the color *C* of the current point *P*(*x*, *y*, *z*). Let *T*(*p*) be the trilinear interpolation result of the volume data point *P*(*x*, *y*, *z*), then *C* can be obtained by inputting the interpolation result *T*(*p*) into the transfer function.

For transmitted light, its illumination complies with the definition in the photon energy equation. We set the Fresnel reflectance at 0 degrees *F*0 = 0, which means no refracted light. The energy equation of the transmitted light can be changed to:

$$\begin{array}{c} L_{CT}=L-L_{I} \end{array}$$ (14)

The fusion rendering equation of point *P*(*x*, *y*, *z*) can be defined as:

$$\begin{array}{c} C_{fusion}\left(x,y,z\right)=C\left(x,y,z\right)*\alpha *\left(L_{interp}\left(x,y,z\right)*dim_{1}+L_{CT}*dim_{2}\right) \end{array}$$ (15)

*dim*_1_, *dim*_2_ are brightness adjustment coefficients, which can be set to fixed values. The fusion rendering equation is the accumulation of ambient and transmitted light, which conforms to physical rules.

Execute the ray-casting algorithm until the accumulated transparency is greater than 1 or the ray passes through the volume data. Accumulate all *C*_*fusion*_(*x*, *y*, *z*) to get the final result color:

$$\begin{array}{c} C_{final}=\sum_{p=1}^{n}C_{fusion}\left(x,y,z\right) \end{array}$$ (16)

## 5 Implement

In summary, The final algorithm flow is shown in the Table 1.

**Table 1:** Using precomputed radiance transfer field in cinematic volume rendering.

1: [Precalculation] Construct a global ambient light field using spherical multiple light sources to simulate low-frequency ambient light.
2: [Rendering] Execute the ray-casting algorithm along the camera’s direction.
3: [Rendering] In the ray-casting algorithm, for points whose transmittance is not 0, use the Ambient Light Interpolation equation to calculate the ambient light from the global ambient light field.
4: [Rendering] Use the transmitted light equation to calculate the transmitted light.
5: [Rendering] Substitute the ambient light and transmitted light into the fusion equation to calculate the color.
6: [Rendering] Superimpose all the colors of the light to get the rendering result.

## 6 Results and evaluation

We use CPU i7-13700, 32G memory, and graphics card RTX4060 for rendering. The resolution of the rendered image is consistent with the original axial data.

### 6.1 Transfer function in precomputation

**Fig 9:**
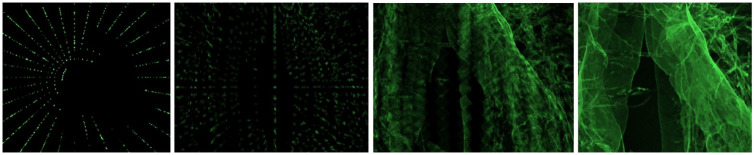
Transparent transfer function precomputed rendering diagram.

**Fig 10:**
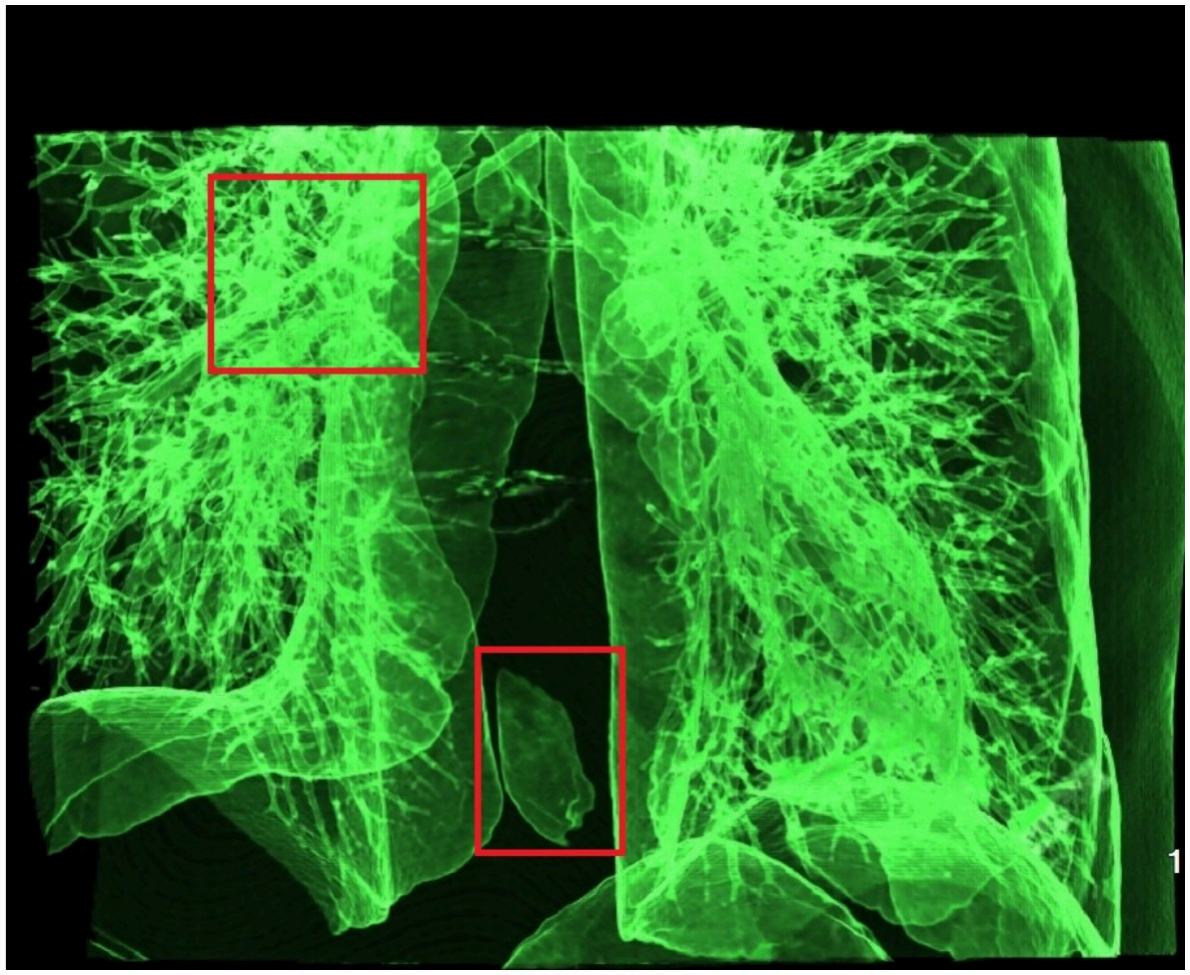
Transparent transfer function precomputed rendering result diagram.

During precomputation, the transfer function is pre-set, and the transmittance of the transfer function affects the precomputation results. The previous Fig 6 shows surface rendering, where the transmittance of the transfer function is 1, resulting in a low-frequency smoothing effect. To verify the precomputation results when the transfer function is transparent, we used a transparent transfer function to precompute separate lung data. By gradually increasing the light source radius, we observed the rendering results. As shown in the Fig 9, as the light source radius increases, the rendered area gradually expands, and the rendering content becomes richer. When the maximum radius is reached, as shown in Fig 10, the voxel is fully rendered. We counted the voxel intensity under different camera angles, finding that the rendered color correlates with the number of visible voxels superimposed from that angle, as indicated by the red-marked areas with varying degrees of overlap. Since we only use the neighborhood around each voxel for calculations during the fusion process, other positions are unaffected, ensuring that the precomputation result remains low-frequency even with a transparent transfer function.

### 6.2 Effectiveness of fusion algorithm

**Fig 11:**
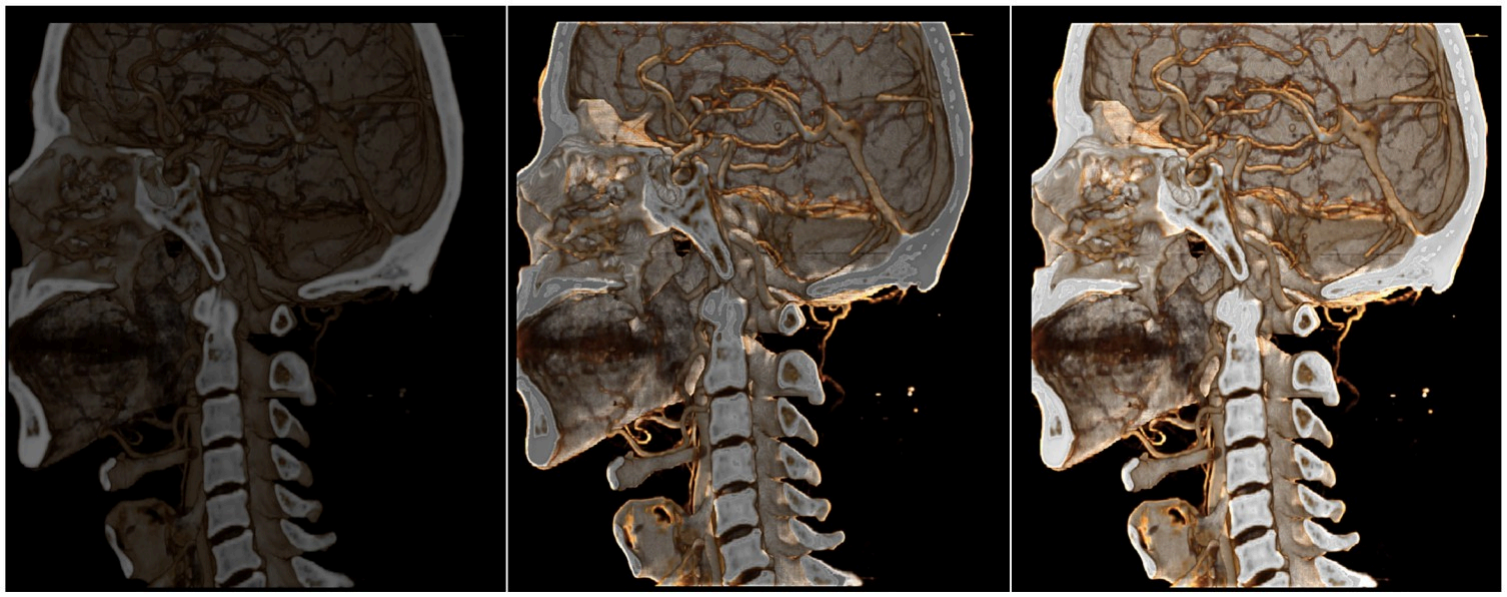
Ambient light and transmitted light comparison diagram.

The fusion algorithm comprises two components: ambient light and transmitted light. Using only ambient light approximates the result of multi-light source path tracing, while using only transmitted light represents a single-ray physical rendering based on the ray casting algorithm. As shown in Fig 11, it is evident that using transmitted light alone does not yield satisfactory results. When only ambient light is used, due to its low-frequency light, the rendering result exhibits low contrast, and the brightness tends to average out when viewed from the camera direction. In contrast, our method improves the display effect by combining transmitted light with ambient light. By superimposing transmitted light from the camera direction onto the ambient light, we enhance the light intensity from that direction, significantly improving both the brightness and contrast of the area affected by transmitted light. This approach effectively enhances the overall rendering quality.

### 6.3 Ray-casting transmitte light

Although refraction can be added to transmitted light, similar to ambient light, this approach has drawbacks. On one hand, it increases computational complexity of real-time rendering; on the other hand, refraction disperses the light intensity in that direction, which may enhance overall brightness but reduce contrast.

**Fig 12:**
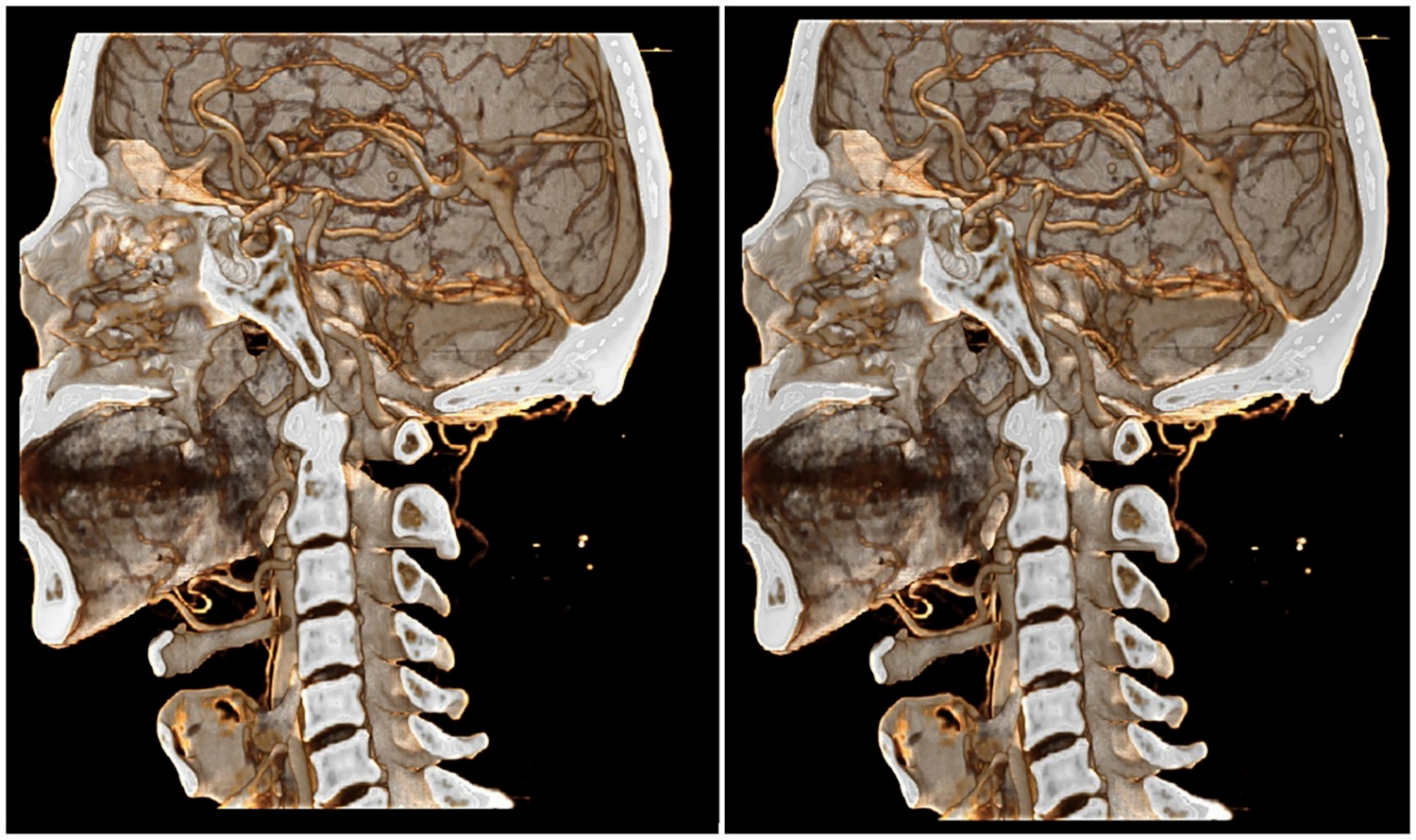
Transmitted light comparison diagram.

As shown in Fig 12, the image on the left, which includes refraction, shows a slight increase in brightness in the brain background area compared to the image produced by our algorithm on the right. However, the overall display effect remains comparable. Therefore, incorporating refraction into the transmitted light has a minimal impact on the rendering result. Our fusion algorithm omits complex refraction calculations, thereby improving computational efficiency without compromising rendering quality.

### 6.4 Efficiency

**Fig 13:**
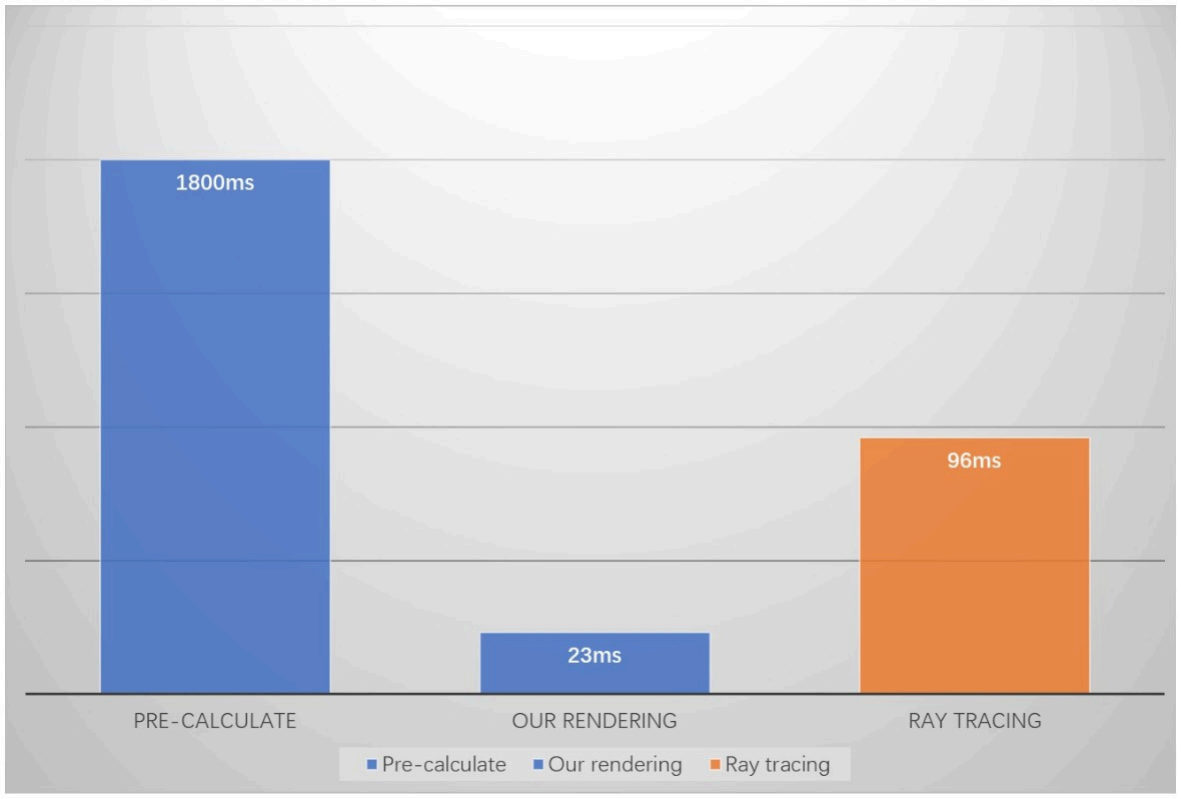
Rendering time diagram.

Our algorithm is divided into two parts: precomputation and real-time rendering. We analyze the rendering time of the algorithm in Fig 11. As shown in Fig 13, the precomputation time constitutes the majority of the total time. Since the precomputation does not affect real-time rendering, our algorithm utilizes these precomputed results during rendering, which reduces the computational complexity and improves efficiency. Consequently, the real-time rendering efficiency of our algorithm surpasses that of the ray tracing algorithm [12].

**Fig 14:**
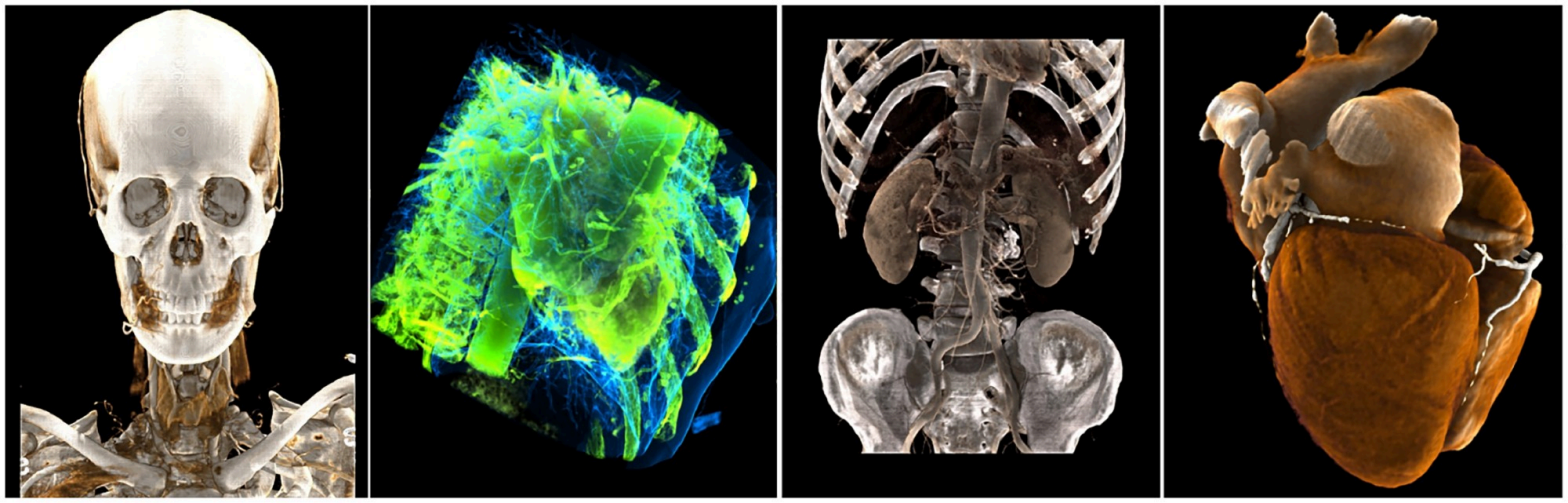
Render dataset diagram.

We have rendered some medical image datasets, As shown in Fig 14 and Table 2. We used the planes where the 72 uniformly distributed points on a sphere lie as light sources for precomputation. To evaluate various transfer functions, we utilized medical imaging data from different body parts and applied the various transfer functions listed in the table for rendering. As shown in the “Precomp.” column of Table 2, although the precomputation time varies slightly due to different transfer functions and the varying sizes of the medical imaging data, all calculations were completed in approximately 2 seconds. This time is acceptable for non-real-time processing. As a result of the precomputation, the real-time rendering algorithm significantly reduced the computation time, as indicated in the “FPS” column of the table, ensuring that rendering efficiency meets real-time requirements. Regarding rendering quality, the precomputation effectively mitigates the issue commonly encountered in conventional physical rendering, where insufficient rendering results in certain regions appearing black. However, because this approach uses only a single ray-casting sample, the smoothness of the rendered image is somewhat reduced.

**Table 2:** Render datasets table.

Dataset	Size(voxel^3^)	Transfer function	Precomp.(ms)	FPS
head	512*512*506	bone	1664	21
cardiac	512*512*256	transparent	1542	19
chest	512*512*520	vessal contrast	2353	32
cardiac2	512*512*344	cardiac	1590	21

### 6.5 Comparison

#### 6.5.1 Compare precalculate ambient light

To compare only the precomputed ambient light, we utilized the CR algorithm [25], known for achieving high-quality physical rendering results through photon mapping in six directions. We integrated our precompute ambient light method into this algorithm and reduced the six-directional photon mapping to three directions.

**Fig 15:**
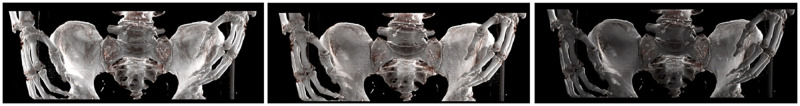
Bone transfer function pelvis render diagram. A: Full Lights Physical Rendering 87ms. B: Half Lights Physical Rendering + Precomputed Ambient Light 48ms. C: Precompute Ambient Light Only 22ms.

**Fig 16:**
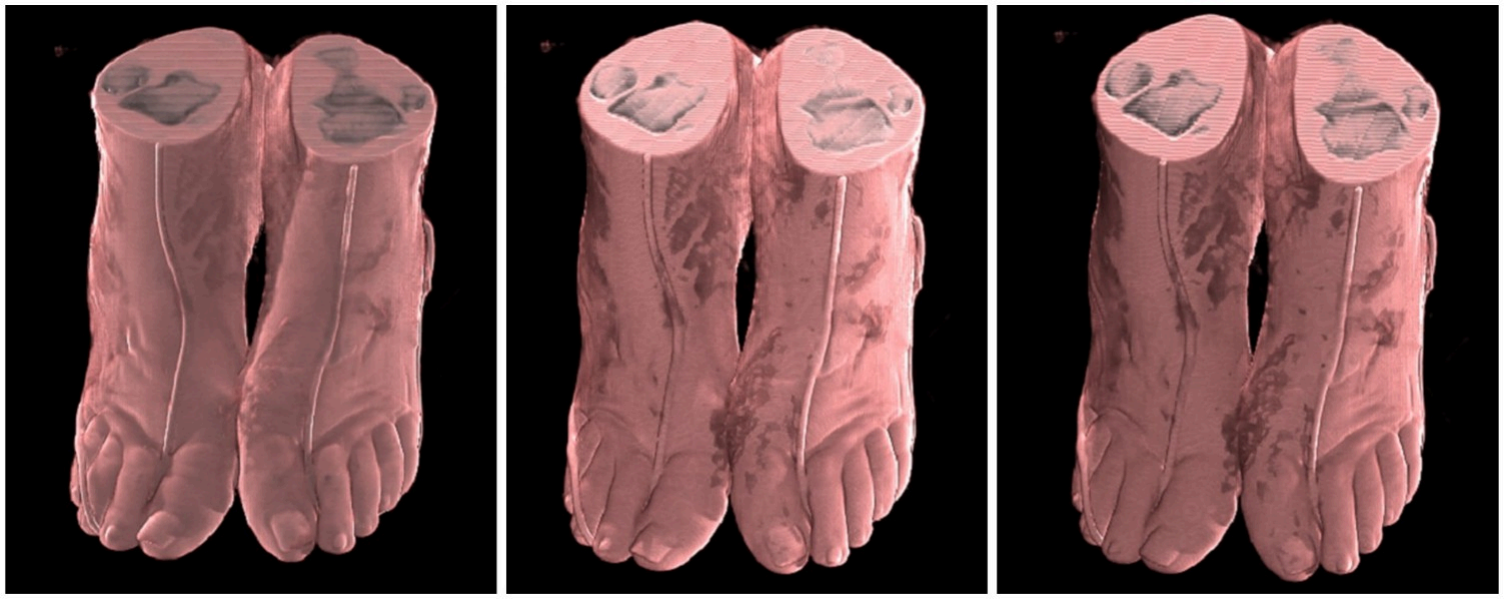
Skin transfer function ankle render diagram. A: Full Lights Physical Rendering 85ms. B: Half Lights Physical Rendering + Precomputed Ambient Light 41ms. C: Precompute Ambient Light Only 25ms.

As shown in Figs 15 and 16, the images from left to right correspond to the CR algorithm [25], the three-directional photon mapping algorithm integrating precomputed ambient light, and the three-directional photon mapping algorithm alone.

Under the bone transfer function, our method effectively enhances the rendering effect, making the output comparable to the original CR algorithm. Under the skin transfer function, while our method strengthens the ambient light effect, it slightly diminishes some direct lighting and shadow effects due of the reduction to three light sources.

Overall, our approach nearly doubles the rendering speed of the CR algorithm [25], with minimal difference in rendering quality, thanks to the reduction of half the light sources and the limited time required for ambient light interpolation.

Ankle and pelvis data from Visible Human Project(VHP) from the University of Iowa(https://medicine.uiowa.edu/mri/facility-resources/images/visible-human-project-ct-datasets).

#### 6.5.2 Compare the whole algorithm

**Fig 17:**
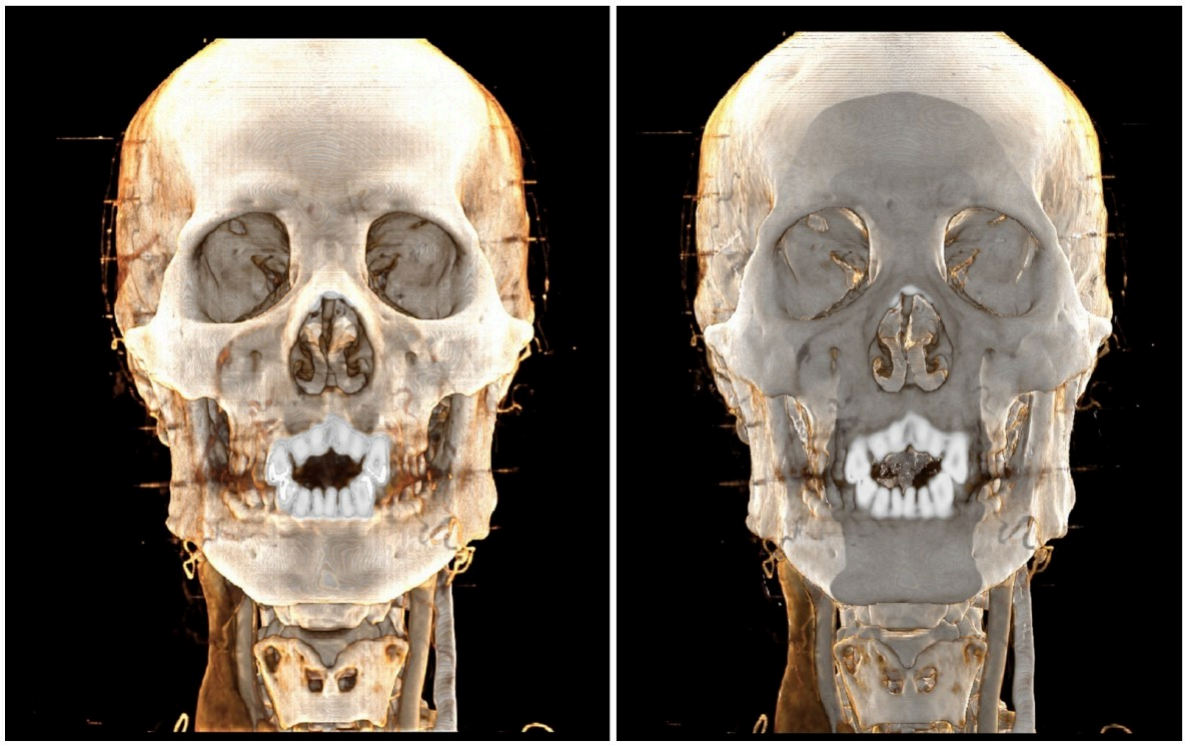
Compare with other algthrom diagram. A:Out Method 21ms. B:Compare Algthrom 85ms.

To compare the overall performance of our algorithm, we used the method proposed by Iglesias-Guitian et al. [16], which achieves higher rendering quality while maintaining a certain level of efficiency. We applied the same lighting conditions for the comparison. As shown in Fig 17, the opponent’s method produces more detailed renderings, while our method delivers stronger shadow effects.

We analyzed the rendering results and found that, in terms of rendering efficiency, our method significantly outperforms the counterpart. In terms of rendering quality, the Monte Carlo-based integration method by Iglesias-Guitian et al. [16] delivers high-quality results when real-time performance is not a priority. If the resolution of the global ambient light field is increased, our method could achieve similar rendering quality, but this would result in a substantial increase in video memory consumption.

Our method can generally achieve physical rendering with effective shadow effects and high-speed efficiency, though the rendering quality is slightly lower.

## 7 Future scope

Our algorithm demonstrates high real-time performance and a good contrast ratio. However, the global ambient light field consumes a significant amount of video memory and requires time for precomputation.

The quality of the global ambient light field is directly proportional to its resolution. Moving forward, we aim to enhance the efficiency and effectiveness of the global ambient light field’s precomputation while optimizing data management and reducing video memory consumption.

## 8 Conclusions

We have developed a precomputed physical rendering algorithm. This algorithm simulates the behavior of shadowless lights by using a spherical arrangement of multiple light sources to precompute lighting for volume data. During the precomputation, we employ unbiased direct lighting. This approach effectively addresses the slow processing speed and noise issues associated with Monte Carlo random sampling, resulting in a uniformly low-frequency illumination in the rendered volume data. By precomputing the ambient light field, our algorithm achieves both acceptable physical rendering quality and optimal rendering efficiency. Additionally, this precomputed ambient light field can be integrated into existing physical volume rendering algorithms to further enhance rendering quality and efficiency.
